# Adding Protein Context to the Human Protein-Protein Interaction Network to Reveal Meaningful Interactions

**DOI:** 10.1371/journal.pcbi.1002860

**Published:** 2013-01-03

**Authors:** Martin H. Schaefer, Tiago J. S. Lopes, Nancy Mah, Jason E. Shoemaker, Yukiko Matsuoka, Jean-Fred Fontaine, Caroline Louis-Jeune, Amie J. Eisfeld, Gabriele Neumann, Carol Perez-Iratxeta, Yoshihiro Kawaoka, Hiroaki Kitano, Miguel A. Andrade-Navarro

**Affiliations:** 1 Computational Biology and Data Mining, Max Delbrück Center for Molecular Medicine, Berlin, Germany; 2 JST ERATO KAWAOKA Infection-induced Host Responses Project, Tokyo, Japan; 3 The Systems Biology Institute, Tokyo, Japan; 4 Sprott Center for Stem Cell Research, Ottawa Hospital Research Institute, Ottawa, Ontario, Canada; 5 Influenza Research Institute, Department of Pathobiological Sciences, School of Veterinary Medicine, University of Wisconsin-Madison, Madison, Wisconsin, United States of America; 6 Institute of Medical Science, Division of Virology, Department of Microbiology and Immunology, University of Tokyo, Tokyo, Japan; 7 Sony Computer Science Laboratories, Inc., Tokyo, Japan; 8 Open Biology Unit, Okinawa Institute of Science and Technology, Okinawa, Japan; 9 Division of Cancer Systems Biology, Cancer Institute, Japanese Foundation for Cancer Research, Tokyo, Japan; University of Chicago, United States of America

## Abstract

Interactions of proteins regulate signaling, catalysis, gene expression and many other cellular functions. Therefore, characterizing the entire human interactome is a key effort in current proteomics research. This challenge is complicated by the dynamic nature of protein-protein interactions (PPIs), which are conditional on the cellular context: both interacting proteins must be expressed in the same cell and localized in the same organelle to meet. Additionally, interactions underlie a delicate control of signaling pathways, e.g. by post-translational modifications of the protein partners - hence, many diseases are caused by the perturbation of these mechanisms. Despite the high degree of cell-state specificity of PPIs, many interactions are measured under artificial conditions (e.g. yeast cells are transfected with human genes in yeast two-hybrid assays) or even if detected in a physiological context, this information is missing from the common PPI databases. To overcome these problems, we developed a method that assigns context information to PPIs inferred from various attributes of the interacting proteins: gene expression, functional and disease annotations, and inferred pathways. We demonstrate that context consistency correlates with the experimental reliability of PPIs, which allows us to generate high-confidence tissue- and function-specific subnetworks. We illustrate how these context-filtered networks are enriched in bona fide pathways and disease proteins to prove the ability of context-filters to highlight meaningful interactions with respect to various biological questions. We use this approach to study the lung-specific pathways used by the influenza virus, pointing to IRAK1, BHLHE40 and TOLLIP as potential regulators of influenza virus pathogenicity, and to study the signalling pathways that play a role in Alzheimer's disease, identifying a pathway involving the altered phosphorylation of the Tau protein. Finally, we provide the annotated human PPI network via a web frontend that allows the construction of context-specific networks in several ways.

## Author Summary

Protein-protein-interactions (PPIs) participate in virtually all biological processes. However, the PPI map is not static but the pairs of proteins that interact depends on the type of cell, the subcellular localization and modifications of the participating proteins, among many other factors. Therefore, it is important to understand the specific conditions under which a PPI happens. Unfortunately, experimental methods often do not provide this information or, even worse, measure PPIs under artificial conditions not found in biological systems. We developed a method to infer this missing information from properties of the interacting proteins, such as in which cell types the proteins are found, which functions they fulfill and whether they are known to play a role in disease. We show that PPIs for which we can infer conditions under which they happen have a higher experimental reliability. Also, our inference agrees well with known pathways and disease proteins. Since diseases usually affect specific cell types, we study PPI networks of influenza proteins in lung tissues and of Alzheimer's disease proteins in neural tissues. In both cases, we can highlight interesting interactions potentially playing a role in disease progression.

## Introduction

The advent of high-throughput techniques to measure and perturb molecular species in a systematic way has enabled researchers to assess the different layers of cellular metabolism under different experimental conditions. Protein-protein interaction (PPI) networks created by a variety of methods including yeast-two-hybrid (Y2H), mass-spectrometry (MS) and computational predictions [1], [2] are valuable research resources, and have been used heavily in the last decade. However, a major drawback of these data is that the artificial expression systems used to reconstruct PPI networks do not take into account two of the many factors that are essential to understand the biology of the cell: first, the time-point at which the proteins are expressed (e.g., cell-cycle or developmental stage) and second, the tissue or intracellular compartment where the proteins are expressed or located (different organs and tissues have very specific protein compositions). Therefore, two proteins may be reported as interaction partners, although they are expressed in different tissues or at different time-points. While high-throughput studies acknowledge these caveats, PPI databases collect these data without mechanisms explicitly directed to discern the biological plausibility of a reported interaction. Therefore, the selection of proteins expressed in a specific cell type or compartment would allow the generation of subnetworks that more realistically represent biological processes in the respective cell types or cellular compartment.

Several attempts have been made to investigate the tissue-specific binding behavior of single proteins and the spatio-temporal dynamics of PPI networks [3], [4], [5], [6], [7], [8]. In a recent study evaluating the characteristics of publicly available PPI databases, we demonstrated that the use of subnetworks (which include only interactions of proteins expressed in the same tissue) identifies potential mechanisms or pathways that would remain obscured if the complete PPI database was used [9].

In addition, many proteins have multiple functions, carried out in cooperation with distinct sets of interacting partners. Networks of interacting proteins with coherent function have been termed context networks [10]. Here, we adopt this notion of context and extend it to PPIs or networks of proteins being expressed in the same tissue or cooperatively transmitting signal flow.

There is a lack of studies testing systematically the potential of adding context information to PPI networks in recovering meaningful PPI subsets and, although there are a few approaches that allow to add expression or functional information to PPI data [11], [12], [13], convenient methods for the creation of such context-specific subnetworks are generally missing.

Here, we introduce an approach to add context to PPI networks using annotations and relations between the interacting partners and demonstrate that context-specific PPI networks are enriched in high-confidence interactions. We use this approach to investigate how the proteins of the human influenza virus interfere with the immune response of the host cell in a tissue-specific manner, finding novel potential regulators of influenza virus pathogenicity, and to study the brain-specific signaling pathways that play a role in Alzheimer's disease, identifying a pathway involving the altered phosphorylation of the Tau protein. Thereby, we illustrate how the addition of context to PPI networks can guide researchers in the discovery of meaningful interactions and pathways, which would otherwise be obscured by the vast amount of irrelevant (for a specific question) and partly erroneous amount of PPI data.

## Materials and Methods

### Data sources

Our approach to add context-specific information to human PPI data was implemented in the HIPPIE database [14]. HIPPIE is an integrated PPI database that currently contains more than 101,000 interactions of ∼13,500 human proteins. HIPPIE is regularly updated by incorporating interaction data from major expert-curated experimental PPI databases (such as BioGRID [15], HPRD [16], IntAct [17] and MINT [18]) in an automated manner using the web service PSICQUIC [19]. All interactions have an associated confidence score based on the sum of cumulative supporting experimental evidence.

Individual proteins were associated with tissues, subcellular locations and biological processes in the following manner. First, proteins were associated with tissues (based on their gene expression profiles retrieved from BioGPS [20] and using the method defined in [9]) or defined as housekeeping (using a list from [21]). Next, associations with biological processes and subcellular locations were determined according to the EBI Gene Ontology (GO) annotation (release from October 28, 2011; reduced to GO slim terms) [22], and to MeSH terms belonging to “Diseases” (class C) or “Tissues” (class A10) that annotate the biomedical references associated to them in MEDLINE (release 2012; gene2pubmed at NCBI ftp site).

### Context association

We associated an interaction with a tissue when both interactors are expressed in the same tissue (e.g. “lung”). Given a term of a functional ontology, we associated an interaction with this function when both interactors are annotated with either the given functional term or with children of it in the hierarchy of the ontology. For example, the GO term “transport” would be associated with an interaction between a protein annotated as involved in “vacuolar transport” and another protein annotated as involved in “nucleocytoplasmic transport”. Functional terms considered were either GO terms or MeSH terms. We excluded the rather unspecific top-level terms ‘biological process’, ‘cellular component’ and ‘cell’. Additionally, we ignored categories that are associated to less than 20 interactions.

### Edge directionality

Our approach includes a method to infer directed PPIs. This inference of interaction (edge) directionality needs sets of proteins predefined as sinks and sources. As default sources and sinks, we connected all proteins annotated with the GO terms ‘receptor’ and ‘sequence-specific DNA binding transcription factor activity’, respectively, in the UniprotKB [23]. This is done assuming that signal pathways follow the transmission of information through interacting proteins starting in cell surface receptors that collect external cues and ending in transcription factors as final effectors on gene regulation, following [24]. To infer edge directionality, all pairwise shortest paths between proteins of the source and the sink sets present in the generated output network are calculated. We do not consider edge weights and, hence we are able to determine each shortest path in linear time via a breadth-first search. An edge of the network is considered to be directed if at least one shortest path goes through that edge. The direction of the path (from source to sink) determines the direction of the edge. Edges with conflicting orientations of passing paths are not assigned directionality.

### Pathway enrichment analysis

For the evaluation of the influenza virus host factor network generation we performed pathway enrichment analysis with ConsensusPathDB (run on August 30, 2012; [25]). We used a cut-off of 0.05 on the q-value, which is the false discovery rate (FDR) adjusted equivalent to the p-value. The background control for the tests was the complete list of proteins annotated as expressed in the given tissues (and with PPI information in HIPPIE).

### Definition of up-regulated genes upon influenza infection

We retrieved the preprocessed microarray data described in [26] measuring gene expression changes over multiple time points in a lung adenocarcinoma cell line (Calu-3) infected with influenza A/Netherlands/602/2009 (H1N1). To select steadily up-regulated genes we filtered for probes differentially expressed at the last three time-points in the time series (30, 36 and 48 h) with a q-value less than 0.01 and a log2 fold change greater than 1.

### Literature mining protocol to obtain PPIs associated to Alzheimer's and protein phosphorylation

To generate a list of PPIs related to Alzheimer's and protein phosphorylation, first, we used the webserver MedlineRanker [27] to retrieve a list of ranked PubMed abstracts (corresponding to manuscripts published within the last 5 years) according to their relevance to the search term “Alzheimer phosphorylation”, which relates loosely to the question of interest. Next, we input the top 50 abstracts from MedlineRanker into the webserver PESCADOR [28], which extracts a network of potential PPIs based on a set of PubMed abstracts. In our example, PESCADOR outputs 10 interaction pairs (type 2; co-occurrence of genes or proteins within a sentence containing a biointeraction term), of which only 4 pairs existed in HIPPIE as scored interactions (PSEN1:PSEN2, GSK3B:MAPT, APP:BACE1, PPP2R4:SET). These confirmed PPIs were then used as input for further analysis.

## Results

### Context-specific and directed PPI networks

We inferred context information for all interactions in the human PPI database HIPPIE [14]. This database collects human PPIs for which there is experimental evidence. The amount and quality of the experimental evidence supporting each PPI is evaluated with a confidence score that ranges from 0 to 1. In a first step, we associated all 13,477 proteins in HIPPIE with the following attributes: tissue-expression, GO biological process and cellular compartment, and inferred annotations for the MeSH categories disease and tissue. We then inferred context associations to the PPIs according to the annotations of the interacting proteins and taking into account the hierarchical structure of GO and MeSH terms (see Materials and Methods for details).

By assuming that a large fraction of signaling events transmits information from proteins sensing environmental changes to effector proteins altering the cellular state, we computed shortest paths from membrane-bound receptors to transcription factors (TF) through the network. From the predicted information flow we assigned edge directionality to interactions on these paths (see Materials and Methods for details).

**Figure 1:**
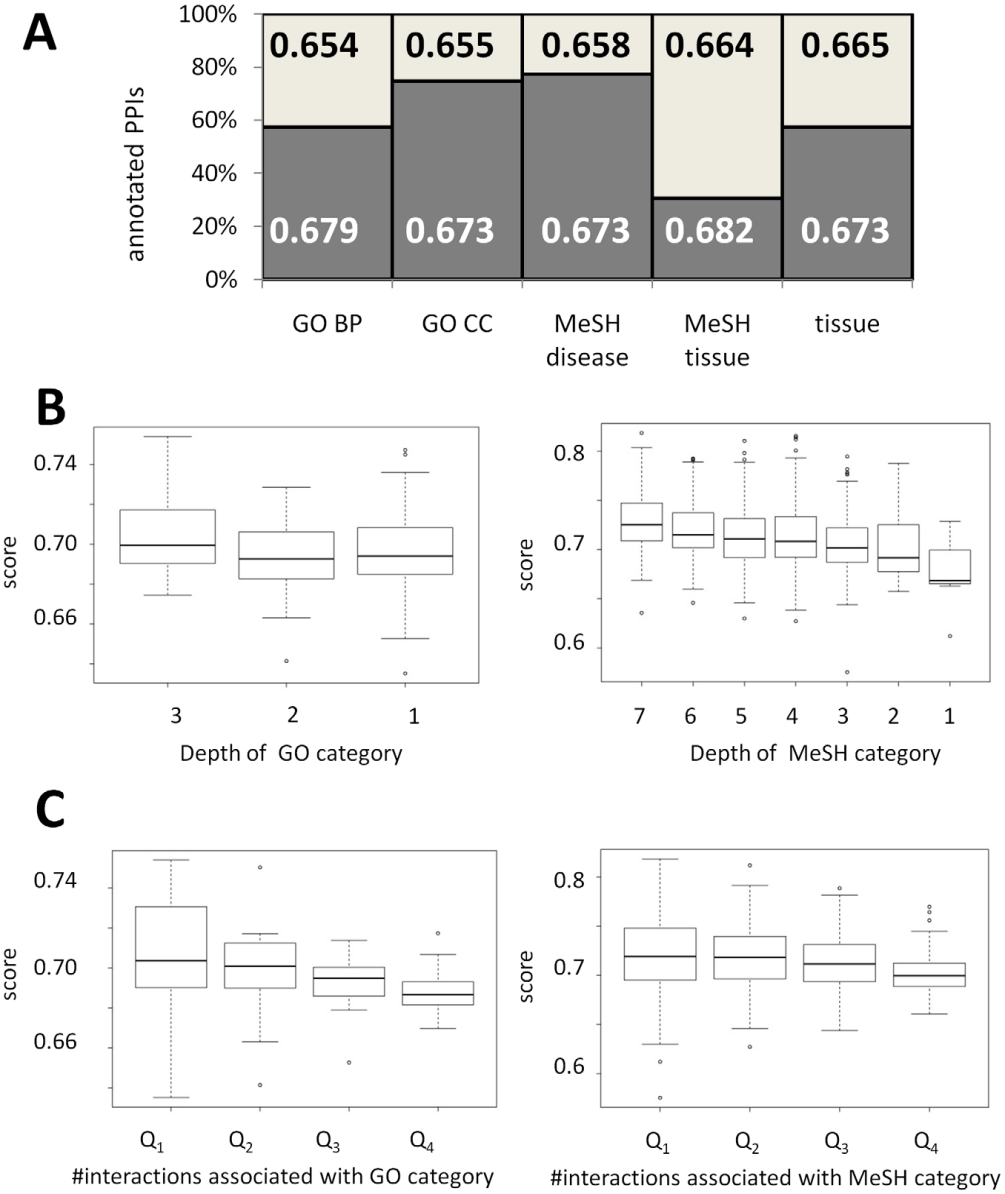
Experimental reliability of annotated PPIs. (**A**) All 101,131 PPIs in HIPPIE are scored according to their associated experimental evidence with a value that ranges from 0 to 1 and increases with the quality and amount of experimental evidence reported in PPI databases [14]. We were able to infer context to a fraction of interactions according to: GO terms biological process (BP) and cellular component (CC), MeSH terms (subcategories disease and tissue) and tissue or housekeeping expression. The numbers in the bars indicate the mean experimental score of the non-annotated fraction (above, black font) and of the annotated fraction (below, white font), respectively. All mean-score differences between annotated and not annotated interactions were significant (p<0.001; Mann-Whitney-test). (**B–C**) Box plots visualizing the distribution of experimental scores of PPIs associated with GO (left) and MeSH (right) term categories. (**B**) The scores for GO and MeSH terms decreased generally for less specific terms (the only exception was GO terms depth 2, which was associated with interactions of a lower mean confidence as compared to GO terms depth 1). (**C**) GO and MeSH terms were subdivided in quartiles according to the number of interactions annotated for each category. The scores decreased for terms associated to higher numbers of interactions.

Overall, we were able to associate context to more than 97,000 of the 101,131 interactions of the current version of HIPPIE. Interactions for which we inferred or collected annotations had significantly better experimental evidence (Figure 1A). This suggests that annotated interactions might have higher biological significance than non-annotated ones.

As expected, we observed that more specific context categories were associated to interactions with higher experimental reliability: while the confidence scores of interactions with rather unspecific and ubiquitous terms resemble the overall confidence score distribution, interactions with highly specific terms usually have a higher than average confidence score (Figure 1B-C). For example, the 43,372 interactions associated with the GO category ‘cytoplasm’ (of depth 1 in the GO hierarchy) have an average confidence score of 0.675 as compared the average of 0.670 over all interactions. On the other hand, the 159 interactions associated with the (depth 3) GO category ‘ribonucleoprotein complex assembly’ have an average confidence score of 0.754. We observed a similar tendency for more specific MeSH terms to have a higher experimental reliability.

To demonstrate that our automated context association approach allows identification of relevant interactions, we tested if networks of interactions of our inferred MESH-based disease-annotation are enriched in well-known disease proteins. Therefore, we repeatedly generated disease-context networks around a set of canonical disease proteins. As a canonical disease protein specification, we retrieved the manually curated UniProt Knowledgebase disease protein annotation. For each of the canonical disease proteins, we generated two types of networks: (a) disease networks consisting only of interaction partners of the disease proteins that we had associated with the equivalent MeSH disease term and (b) unfiltered PPI network consisting of all interaction partners of the disease protein from HIPPIE. We did this for all disease proteins where the disease was associated with at least two disease proteins in UniProt and at least two interactions that we had associated with this disease. To quantify the enrichment of disease proteins in these networks we repeatedly calculated the F1 score, the harmonic mean of precision and recall (F1 = 2*precision*recall/(precision+recall)). A one-sided Mann-Whitney-test comparing the distribution of F1 scores between the disease networks and the non-filtered networks indicated that the F1 scores for the disease networks were significantly larger (p<0.05) proving an enrichment of disease proteins in the disease filtered networks (without losing sensitivity by removing disease proteins in the filtering step). The mean precision on the filtered networks was 0.47 and on the unfiltered networks 0.21. The mean recall for the filtered networks was 0.14 and for the unfiltered networks 0.15. This illustrates that in exchange for a small decrease in recall the precision can be more than doubled by applying the MeSH disease filter.

We then investigated the potential of edge directionality inference based on the shortest paths between membrane-bound receptors and TFs through the PPI network to recover known pathways. We retrieved pathway annotations (extracted from WikiPathways download March 29, 2012) and computed the shortest paths through HIPPIE between all pairs of receptors and TFs within the same pathway (excluding only pairs that directly interact or could not be connected by any path). We counted the number of proteins of each pathway found on the shortest paths. We found for 3163 of the 5063 pairs that this approach correctly identified proteins of the selected pathway. The mean precision (the fraction of proteins on the paths that indeed belonged to the correct pathway) over all combinations of receptors with transcription factors was 0.20. The mean recall (the fraction of the pathway that was recovered by considering the paths between one receptor and one transcription factor) was 0.02.

To assess if the agreement between shortest paths and canonical pathways was larger than expected by chance, we generated a background distribution by computing repeatedly the shortest paths between a receptor and a TF from different pathways and computed the overlap between the proteins on the shortest paths to either the TF- or the receptor-containing pathway. We found that the overlap distribution was significantly higher when the receptor and the TF were members of the same pathway (p<0.001; Mann-Whitney-test) proving the potential of shortest paths to recover the signal flow between TFs and receptors when functionally related pairs of receptors and transcription factors are chosen.

We wondered if we could further increase the overlap between the shortest paths and the canonical pathways by filtering the networks for tissue expression. To associate pathways with tissues, we determined for each pathway which tissues were enriched among the genes of the pathway (Supplementary Table S1 lists pathway that are associated to more than 2-fold enriched tissues). Inspection of the tissues enriched among proteins forming a pathway revealed that in many cases they indeed reflect plausible locations for pathway activity. For example, immune response pathways were enriched among blood cells and pathways associated with neurodegenerative diseases and addiction in brain-related tissues.

We repeated the computation of shortest paths linking receptors to transcription factors in tissue-specific networks for combinations of pathways and tissues listed in Supplementary Table S1 and for all pairs of receptors and transcription factors that were expressed in the respective tissue. Indeed, we observed an increase of the mean precision to 0.24, which indicates that we could increase the amount of meaningful interactions by additionally filtering for tissue expression. The recall remained low (at 0.03), which is not surprising since many pathway-related proteins were not present in the considered tissue-specific networks and, hence, could not be detected. Again, the amount of pathway proteins on the tissue-specific shortest paths between receptors and TF from the same pathway was significantly larger as compared to shortest paths between receptors and TF from different pathways (p<0.05).

To further investigate if the described context-associations can help to extract pathway information from networks, we compared the frequency of protein pairs being member of the same pathway (as defined by WikiPathways) among tissue-specific PPIs (both proteins where required to be co-expressed in at least one tissue) and compared this frequency to PPIs between proteins that are not expressed in the same tissue. We observed that interacting protein pairs that are expressed in the same tissue are indeed more likely to be in the same pathway as compared to interacting protein pairs that are expressed in disjoint sets of tissues (p<0.001). This, again, demonstrates that the annotations have captured properties related to pathways and suggests that the filtering helps revealing pathway information.

In the next sections we use the context-associated PPI network to obtain novel insights into the mechanisms of human disease: we perform a targeted study of the PPI network surrounding the human proteins that interact with influenza virus proteins to find potential regulators of viral pathogenicity, and we explore the question of whether and how altered protein phosphorylation might be a cause of Alzheimer's disease.

### Context-specific subnetworks of influenza virus host factors identify known disease pathways and suggest novel pathogenesis mechanisms

**Figure 2:**
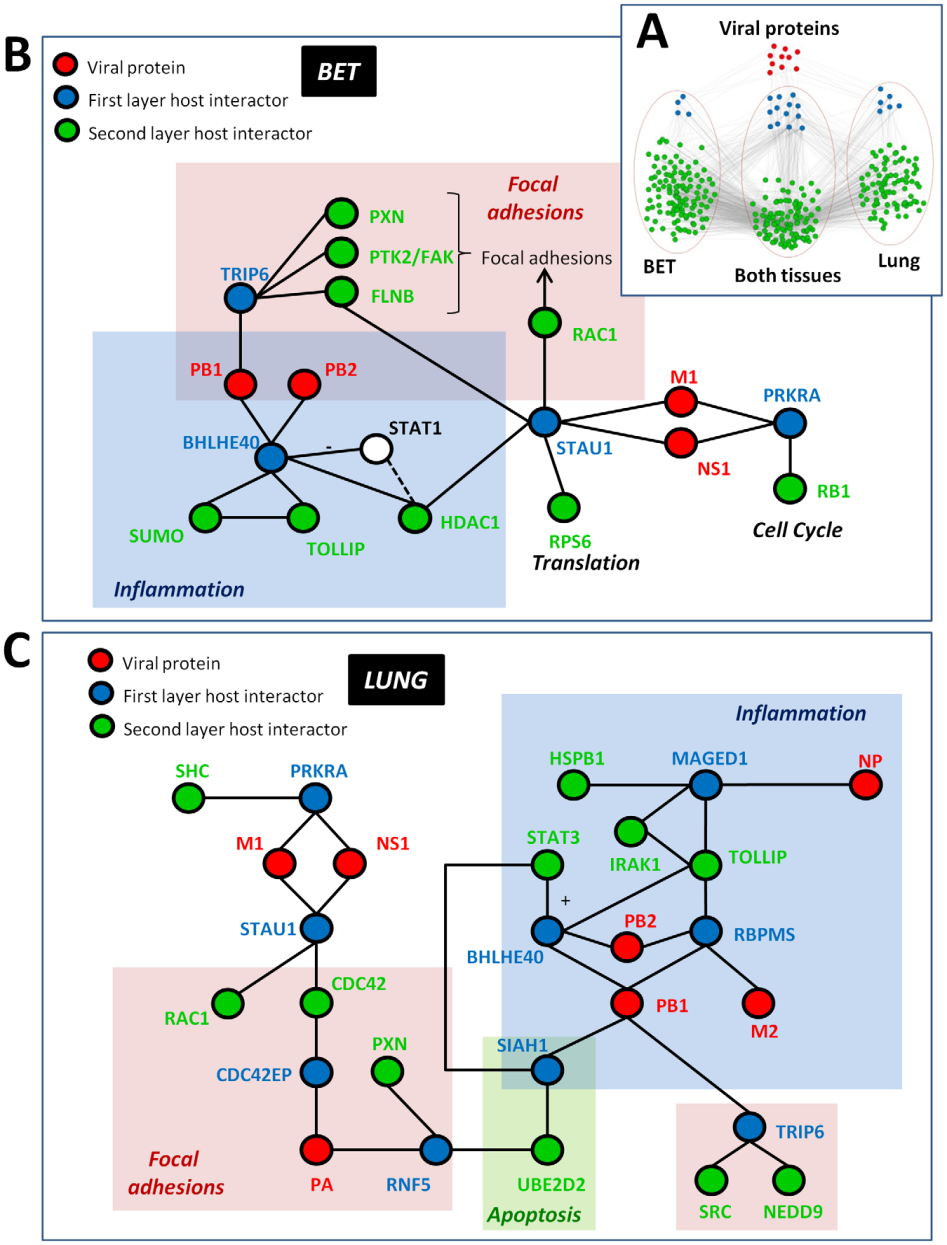
Tissue-specific PPI subnetwork of human proteins interacting with influenza virus proteins. (**A**) Influenza proteins (red) interact with 23 ‘first layer’ host proteins (blue). These first layer proteins have interaction partners that are specific for the bronchial epithelial tissue (BET) subnetwork, for the lung subnetwork or are shared between both subnetworks (all in green). Details of the genes in this figure are given in a Cytoscape file (File S1). (**B and C**) Mini-networks for BET (B) and lung (C) were created from tissue-specific protein networks linking viral proteins to host proteins whose transcript was up-regulated after influenza virus infection (for a complete list of interactions, see Supplementary Table S4). Viral protein nodes are shown in red, first layer host interactors in blue and second layer host interactors in green. The STAT1 protein (shown in (B) as the white node) was not one of the original network-derived nodes, but was included due to its association with two other network nodes (BHLHE40 and HDAC1) and its known role in mediating inflammation in response to viral infection. General functions associated with different areas in each mini-network (e.g., ‘Inflammation’ and ‘Focal adhesions’) are described by partially transparent colored boxes in both (B) and (C).

We analyzed PPI data of human proteins that interact with influenza virus proteins. Influenza viruses infect bronchial epithelial tissue and many cell types in the lung, sometimes resulting in viral pneumonia [29]. We started by obtaining a list of 87 human proteins that have been shown to interact with at least one influenza virus protein in a previous study [30]. From this list, we observed that 23 proteins were expressed in bronchial epithelial tissue (BET), in whole lung, or in both tissues - we refer to these proteins as first layer host factors. We created the second layer by filtering tissue-specific proteins (expressed in BET or whole lung) that interact with members of the first-layer (Figure 2A). Together, the first and second layers compose the tissue-specific PPI subnetworks.

Next, we identified known pathways enriched in the BET- and lung-specific PPI subnetworks, and found both similarities and differences in the cellular functions of each (see Materials and Methods for details on the enrichment analysis and a full list of enriched pathways in Supplementary Table S2). Both subnetworks showed enrichment for processes related to programmed cell death and eukaryotic translation. These results are consistent with functions known to be activated or disrupted by influenza virus infection [31], [32], [33]. In addition, proteins in the BET subnetwork exhibited a stronger signature in processes involved with transcriptional regulation, sumoylation, and the regulation of mRNA stability (in particular, the stability of AU-rich element-containing mRNAs). Although these processes tend to be associated with general housekeeping functions, we point out that many cytokine and interferon mRNAs contain AU-rich elements [34]. This observation suggests, hypothetically, that influenza virus proteins may function to dysregulate cytokine mRNA stability in BET, a function that could impact influenza virus pathogenesis through modulation of immune cell infiltration and function. In relation to sumoylation, it has been noted recently that influenza virus can gain protein functionality during infection by interacting with the sumoylation system of the host cell [35]. On the other hand, the lung subnetwork was uniquely enriched for processes related to cell-substrate adhesion (pathway “signaling events mediated by focal adhesion kinase”). Because cell adhesion is important for maintaining cellular viability and epithelial barrier function, it is possible that influenza virus protein-mediated interference with this process could impact both the amount of virus-inflicted damage upon the lung and dissemination of influenza virus into extra-pulmonary sites.

Cells respond to influenza infection by producing cytokines and chemokines [36], [37], while viral proteins counteract this innate immune response. One example of a viral protein that directly interferes on the protein level with cellular immune pathways is NS1 (its involvement in immune response suppression is reviewed in [38]). Here, we noted that the lung PPI subnetwork – which was centered on viral protein-host protein interactions – was enriched for several curated pathways involving Toll-like receptor (TLR) and IL-1 receptor (IL-1R) signaling (e.g., “TLR JNK”, “TRAF6 mediated IRF7 activation in TLR7/8 or 9 signalling”, “IL-1 JNK”, “TLR ECSIT MEKK1 JNK” and “IL1-mediated signaling events”). Although these pathways are expected to be activated in response to viral infection, no previous study has identified any role for any influenza virus protein in perturbing TLR or IL-1R signal transduction. Several host proteins were consistently observed in most/all of the enriched TLR/IL-1R pathways from the influenza PPI lung subnetwork, including IRAK1, TOLLIP and MyD88. Under normal conditions, the IRAK1 kinase associates with TOLLIP (an inhibitory molecule), and upon receptor stimulation, IRAK1 is recruited to the TLR/IL1R-receptor complex through its interaction with MyD88 (reviewed in [39]). Recruitment results in activation of IRAK1 kinase activity and subsequent activation of MAP kinase pathways, NF-κB-dependent gene expression and interferon α induction. Altogether, these observations suggest the novel possibility that influenza virus proteins interfere with TLR/IL-1R signaling in lung – possibly by accessing a critical regulator of TLR/IL-1R signal transduction (i.e., IRAK1) – an observation that may have implications for the regulation of pathogenesis associated with influenza virus infections.

A recent study demonstrated that signaling through the IL-1 receptor has a protective effect in mice infected with the pandemic 1918 influenza virus [40]. Another study reported that IL-1 receptor-deficient mice succumbed more easily than wild-type mice to infection with an H5N1 virus of low pathogenicity (A/Hong Kong/486/1997) [41]. Moreover, IL-1 receptor-deficient mice showed reduced inflammatory pathology upon infection with A/Puerto Rico/8/34 (H1N1) influenza virus [42]. Several studies also established that influenza virus infection is sensed by TLR7 in plasmacytoid dendritic cells [43], [44], [45], [46], [47], [48]. However, none of these studies addressed the significance of IRAK1 in influenza virus pathogenicity. Our study thus exemplifies how our network analysis can identify potential regulators of influenza pathogenicity for experimental testing, for example, by assessing influenza virus infections in IRAK1-deficient cells or mice.

Next, we aimed to predict more specific novel interference mechanisms by constructing directed and tissue-specific protein networks linking the viral proteins with proteins whose corresponding transcript was up-regulated after influenza virus infection. We selected steadily up-regulated transcripts from a microarray experiment measuring gene expression changes over time in a lung epithelial cell line infected with a 2009 pandemic H1N1 virus [26] (228 transcripts were selected in total; see Materials and Methods for more details). As expected, all ten most strongly enriched known pathways among the selected transcripts were involved in infection and the immune response. For example, the most highly overrepresented pathway was interferon alpha-beta signaling (p<10e-20).

We constructed BET- and lung-specific networks connecting the viral proteins with the 228 up-regulated factors by shortest paths. From the shortest paths we assigned directions to the edges on these paths. The directed networks consisted of 577 (BET) and 1056 (lung) PPIs. To examine if these networks might reveal relevant information on how viral proteins interfere with the cellular immune response, we tested for enrichment of known pathways in the directed networks. We found that the directed networks were strongly enriched in immune response-related pathways (especially cytokine-related) even after excluding the 228 up-regulated transcripts, indicating that enrichment was independent of the high fraction of immune response factors in the transcriptomics data (Supplementary Table S3). For example, we observed a significant enrichment in both the directed BET- and lung-specific networks for proteins related to IL-2 and IL-6 signaling and focal adhesions (q-values<0.05). This suggested that we, indeed, might have captured relevant crosstalk between the viral proteins and immune pathways. The full networks are included in the File S1.

To mine the directed networks for interactions that are involved in interference mechanisms of the viral proteins with the cellular immune response, we concentrated, again, on layer one and two host factor proteins on the shortest paths. From the list of curated pathways enriched in both the BET and the lung directed networks (Supplementary Table S3), we selected several cytokine-related pathways (marked in Supplementary Table S3) and filtered for interactions where the second layer protein was in one of these pathways but the layer one protein was not (to specifically detect novel, indirect interference mechanisms). This resulted in a comprehensive BET network consisting of 49 interactions and a lung network formed by 67 interactions including viral proteins and host factors up to layer two (see Supplementary Table S4 for the comprehensive networks and Figure 2 for a manually curated subset of these networks).

Close inspection of these comprehensive cytokine-related networks in both BET and lung revealed several points of potential viral protein-mediated interference with inflammatory pathways (Figure 2). For example, the BET network showed interactions between viral polymerase complex proteins (i.e., PB1 and PB2) and BHLHE40, a transcriptional regulator that cooperates with HDAC1 to repress STAT1 activity [49] (Figure 2B). STAT1 is essential for the activation of interferon stimulated genes, which repress viral replication, and while influenza virus has an established ability to impair STAT1 [50], no such function has been assigned to any of the viral polymerase complex subunits. BHLHE40 also interacts with TOLLIP, a suppressor of TLR signaling [51] (see also the discussion of lung-specific inflammatory pathways above). This implies that the BHLHE40 protein could act as an important access point for influenza virus-mediated interference with host antiviral and inflammatory regulation in BET, and further that viral polymerase subunits may have an important – yet unappreciated – role in this activity.

As in BET, lung-specific cytokine-related networks revealed that influenza virus proteins interface with TOLLIP (Figure 2C). However, it is notable that, in lung, this interaction occurs through BHLHE40 and two additional routes (i.e., MAGED1 and RBPMS), potentially involving up to four viral proteins: (i) the aforementioned polymerase complex subunits, PB1 and PB2; (ii) the viral ion channel protein, M2; (iii) and the viral RNA-binding nucleoprotein, NP. Thus, access to TOLLIP might be particularly important in lung. The PB1/PB2-BHLHE40 interaction is maintained in this tissue type, although the nature of the interaction may differ compared to BET. Specifically, BHLHE40 may favor interaction with STAT3 (Figure 2C), and previous evidence indicates that BHLHE40 stimulates STAT3 activity rather than inducing inhibition [52]. Thus, analysis of context-specific PPIs – in combination with influenza virus-induced changes in the cellular transcriptome – reveal important, putative tissue-specific differences in the ability of viral proteins to interact with cellular immune response signaling networks. Additional experiments will be necessary to further establish the functions of these interactions.

### Search for phosphorylation-dependent PPIs related to Alzheimer's

Assuming no prior expert knowledge on a given topic, we applied a systematic protocol which can, in principle, be used to interrogate the PPI network about the involvement of protein interactions in a complex biological question according to current knowledge. In general, altered states of protein phosphorylation affect the PPI network and can lead to pathogenesis. Our goal in this example was to investigate the possible role of protein phosphorylation in Alzheimer's disease (AD), the most common form of dementia. AD is a degenerative disease manifesting in the brain, and its cause has been hypothesized to be the formation of protein aggregates leading to neuron death, in particular related to the abnormal phosphorylation of the microtubule-associated protein tau [53].

**Figure 3:**
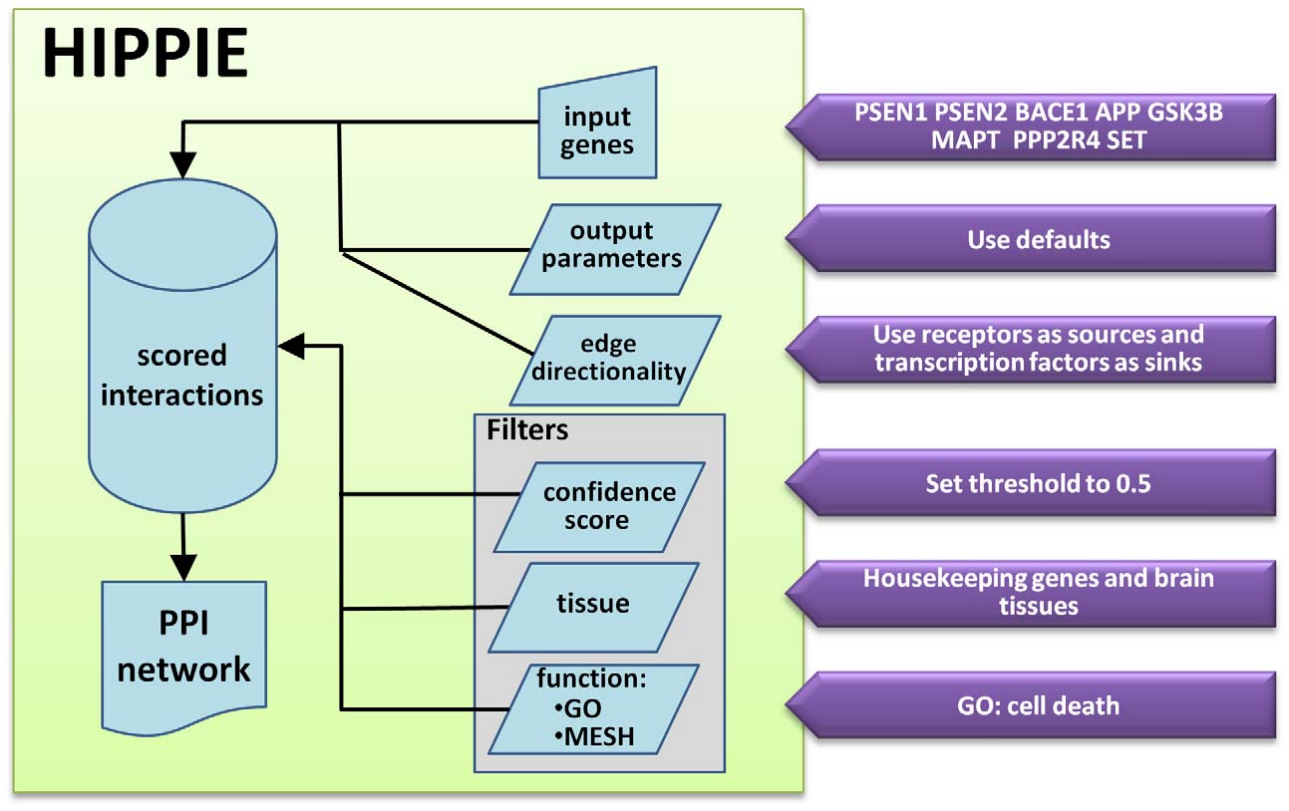
Protocol for extraction of a PPI subnetwork related to phosphorylation in Alzheimer's disease. The flowchart illustrates the input terms and options used to generate a topic-focused PPI subnetwork. Eight genes were selected as a result of an unbiased literature mining query for proteins related to Alzheimer's disease (AD) and phosphorylation (see main text for details). The PPI network of first neighbours of these genes in HIPPIE was generated. Then, filters were applied to focus on a PPI subnetwork or proteins expressed in the brain and related to cell death, thus relevant to AD.

First, we need to input a list of proteins related to the topic. Using a literature mining protocol (see Materials and Methods for details) we generated a list of PPIs related to Alzheimer's and protein phosphorylation: PSEN1:PSEN2, GSK3B:MAPT, APP:BACE1, and PPP2R4:SET. We then studied the network surrounding these interactors (Figure 3).

**Figure 4:**
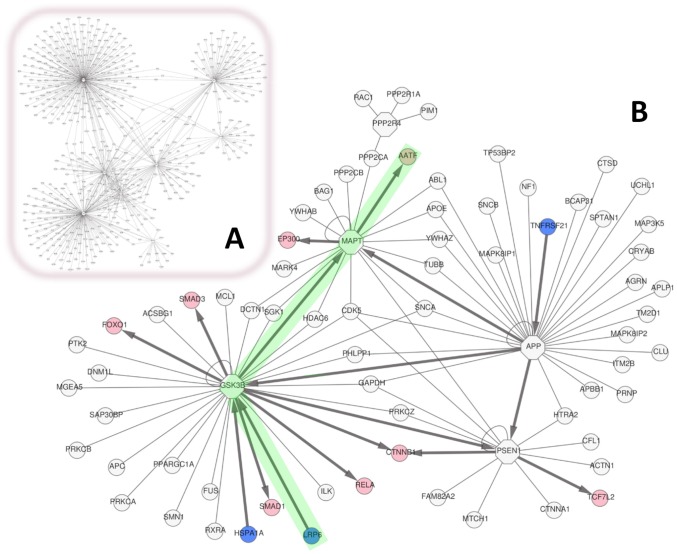
Filtering and highlighting a PPI subnetwork related to phosphorylation in Alzheimer's disease. A PPI network was generated as explained in Figure 3 starting with 8 genes relevant for Alzheimer's disease (AD) and phosphorylation. (**A**) The PPI network contains 727 interactions. (**B**) Filtering for interactions between partners that are housekeeping or expressed in the brain (“whole brain” and “prefrontal cortex”), relate to the GO term “cell death”, and with experimental scores above 0.5, results in a much more focused subnetwork involving 6 of the 8 genes used as input (octagonal nodes). Nodes corresponding to receptors and transcription factors are colored (blue and pink nodes, respectively). Edge directed path analysis from receptors to transcription factors resulted in the association of directionality to some of the edges (arrows). The path LRP6-GSK3B-MAPT-AATF is highlighted in green and described in the text.

The initial PPI network contained 727 interactions (Figure 4A). Interactions could be further filtered on the basis of reasonable criteria, namely by tissue filtering for housekeeping and genes expressed in the brain (we selected “whole brain” and “prefrontal cortex”), and filtering for genes related to the GO term “cell death”, reflecting that AD is characterized by death of neural cells (Figure 4B). Finally, to reveal potential signal transduction pathways we used the inference of edge directionality from receptors to TFs described above (Figure 4B).

Within the resulting network, we highlighted the following path (Figure 4): LRP6-GSK3B-MAPT-AATF. The low density lipoprotein receptor-related protein 6 (LRP6) interacts with glycogen synthase kinase 3B and attenuates the kinase's ability to phosphorylate microtubule associated protein tau (MAPT) [54]. Tau protein can contribute to AD in different ways: 1) the hyperphosphorylation of tau protein can affect microtubule stability, leading to a disassociation of tau protein from the microtubule, possibly followed by the aggregation of phosphorylated tau into neurofibrillary tangles, which are observed in the brains of AD patients [55]; 2) mediated by protein phosphatase 1 and GSK3 activity, Tau filaments interfere with axonal transport in the neuron, which is consistent with deficiencies in axonal transport in AD [56]. Tau protein has been found to co-localize in the cytoplasm with Che-1 (AATF), which is an evolutionarily conserved RNA polymerase II binding protein that accumulates in the cell upon DNA damage [57]. It appears that Che-1/Tau proteins dissociate during neuronal cell death [58]; however, the function of Che-1 in the cytoplasm is unclear, as Che-1 is a nuclear protein that is involved in gene regulation of E2F1 targets and p53 and has pro-proliferative and anti-apoptotic functions [59]. Together, these interactions suggest a complex interplay whereby the Tau phosphorylation state and structure, and context-dependent protein distribution within the cell may contribute to neuronal cell death and AD pathology. An unbiased search for protein phosphorylation in relation to cell death in AD pointed us to this interesting pathway.

## Discussion

The incorporation of tissue-specific expression information to create PPI subnetworks is a useful method to elucidate biological processes that cannot be observed when using the complete PPI network. Here we have shown an approach for the inference of associated context for PPIs based on the annotations of the interacting partners, which enhances the relevance of the annotated interactions. Interactions between proteins expressed in the same location (e.g. lung) or at the same time or developmental stage (e.g. embryo development) can then be selected. Directed pathways can be inferred and highlighted in the filtered network according to sets of sources and sinks corresponding to receptors and transcription factors. Using this approach we were able to identify novel, tissue-specific interactions between influenza virus proteins and cellular inflammatory signaling pathways that may regulate pathogenesis associated with infection, and to describe a brain-specific protein phosphorylation pathway relevant for Alzheimer's disease.

Several methods exist to create subnetworks of the human interactome based on context criteria. For example, POINeT [11] integrates the major PPI databases and allows the creation of tissue-specific networks. To our knowledge we are the first to combine edge directionality, gene expression and functional information for the detection of meaningful interactions. Some approaches exist that infer information flow in a network from the shortest paths (or ‘lowest costs’ if costs are associated with edges) that connects a set of source nodes with sink nodes. Cytoscape plug-ins such as BisoGenet [60] and GenePro [61] find the shortest paths between nodes of the gene and protein network and represent properties of the nodes. SPIKE [62] includes curated pathway data and also calculates pathway inference. The task of identifying signaling events from PPI data and functional protein annotation alone has been addressed in several studies [24], [63], [64] and implemented in tools (e.g. ANAT [65]). Here, we proposed a protocol for edge directionality prediction based on calculating the shortest paths between sources and sinks. This protocol is runtime-efficient, which allowed us to provide it as a web tool that is the first to combine both PPI analysis for inference of edge directionality and PPI filtering by tissue and function (available from http://cbdm.mdc-berlin.de/tools/hippie/).

In summary, we have presented and made available an approach to associate context to PPI networks, which provides novel biological insight into mechanisms of disease. The continuing generation of PPI data and further incorporation into databases, and an increasing quality of annotations attached to genes and proteins will result in further improvements of our methodology.

## Supporting Information

File S1 Network of first and second layer host factors (Figure 2A) in Cytoscape format.
(ZIP)

File S2 Directed BET and lung specific networks connecting first layer viral interactors with upregulated host proteins in Cytoscape format. In the directed network, sources and sinks are color encoded (viral are red and upregulated proteins brown). Cytokine-related proteins are shown as circles.
(ZIP)

Table S1 Tissues more than two-fold enriched among proteins in pathways.
(XLS)

Table S2 Pathways enriched in first and second layer influenza host factor networks.
(XLS)

Table S3 Pathways enriched among directed networks connecting viral proteins with gene products upregulated upon influenza infection.
(XLS)

Table S4 Comprehensive BET and lung PPI networks connecting viral proteins with cytokine-related second layer proteins on shortest paths between viral proteins and gene products upregulated upon influenza infection.
(XLS)
